# Application of DSO algorithm for estimating the parameters of triple diode model-based solar PV system

**DOI:** 10.1038/s41598-024-53582-3

**Published:** 2024-02-16

**Authors:** P. Ashwini Kumari, C. H. Hussaian Basha, Rajendhar Puppala, Fini Fathima, C. Dhanamjayulu, Ravikumar Chinthaginjala, Faruq Mohammad, Baseem Khan

**Affiliations:** 1 School of Electrical and Electronics Engineering, Reva University, Bangalore, India; 2 NITTE Meenakshi Institute of Technology (Autonomous), Bengaluru, India; 3 School of Engineering, DY Patil International University, Akurdi, Pune, India; 4 Mar Baselios Christian College of Engineering & Technology, Thiruvananthapuram, Kerala India; 5 School of Electrical Engineering, Vellore Institute of Technology, Vellore, India; 6 School of Electronics Engineering, Vellore Institute of Technology, Vellore, Tamil Nadu India; 7 Department of Chemistry, College of Science, King Saud University, P.O. Box 2455, 11451 Riyadh, Kingdom of Saudi Arabia; 8 Department of Electrical and Computer Engineering, Hawassa University, 05 Hawassa, Ethiopia

**Keywords:** Energy science and technology, Engineering

## Abstract

Solar Photovoltaic (SPV) technology advancements are primarily aimed at decarbonizing and enhancing the resiliency of the energy grid. Incorporating SPV is one of the ways to achieve the goal of energy efficiency. Because of the nonlinearity, modeling of SPV is a very difficult process. Identification of variables in a lumped electric circuit model is required for accurate modeling of the SPV system. This paper presents a new state-of-the-art control technique based on human artefacts dubbed Drone Squadron Optimization for estimating 15 parameters of a three-diode equivalent model solar PV system. The suggested method simulates a nonlinear relationship between the P–V and I–V performance curves, lowering the difference between experimental and calculated data. To evaluate the adaptive performance in every climatic state, two different test cases with commercial PV cells, RTC France and photo watt-201, are used. The proposed method provides a more accurate parameter estimate. To validate the recommended approach's performance, the data are compared to the results of the most recent and powerful methodologies in the literature. For the RTC and PWP Photo Watt Cell, the DSO technique has the lowest Root Mean Square Error (RMSE) of 6.7776 × 10^–4^ and 0.002310324 × 10^–4^, respectively.

## Introduction

Reduced supply of conventional fuels evidenced by the depletion of petroleum resources has diverted researcher’s attention toward green energy sources (GES). Growing energy demands need an effective and robust technology that can harvest untapped energy resources with minimal environmental impacts^1^. SPV being one of such promising sources, drives the research and development towards energy crisis in terms of cleaner, renewable, and maintenance-free power generation. SPV possesses a drastic surge due to technological developments. To obtain adept and precise control, the SPV cell has to be mathematically modeled^2^. Modeling involves deducing circuit equivalent equations that can trace the PV voltage and current characteristics. Researchers in the literature compromise in terms of complexity by excluding the effect of a few parameters during modeling.

Literature refers to three significant models namely one, two, and three diode models. Single and two-diode models are widely used as they can precisely estimate system behavior^3–6^. One diode model is a simple approach where 5 parameters of the SPV are obtained. This method yields less accuracy in estimating the parameters during varying climatic conditions^7^. To address these drawbacks two diode model consisting of 7 parameters that include the effect of recombination is considered^8^. Due to the increased no of parameters, this approach is complex and time-consuming. These models do not assure the accuracy of the estimated parameters as the effects of recombination are not considered. To overcome this demerit an extra diode is included in parallel which aids in accomplishing the flaws associated with earlier methods^9^.

**Figure 1:**
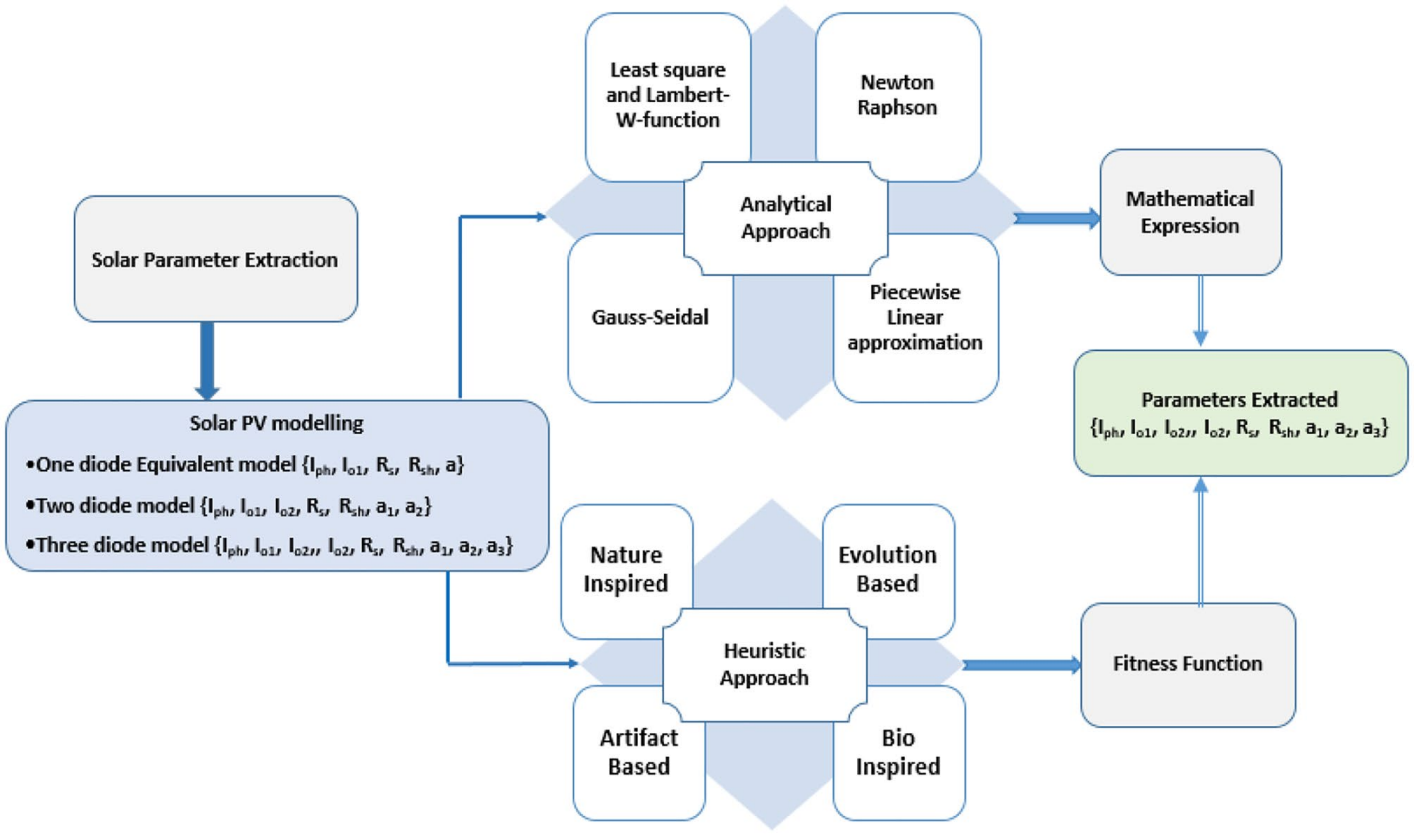
Parameter extraction methods and modeling approaches.

The Fig. 1. Shows the classification of the modeling techniques and the corresponding estimated parameters. The performance of PV systems can be improved using precise and accurate modeling. P–V and I–V curves are the basic performance indicators of the system. Precise and reliable estimation of intrinsic parameters are the prerequisites for implementing an accurate equivalent circuit model. The data provided by the manufacturer in the Datasheet do not reveal complete information of all the intrinsic variables. Estimating accurate information on all intrinsic variables under any climatic condition is the scope of this work. These intrinsic variables change with perturbation in radiation, temperature soiling effects, aging factor, and partial shading^10^.

Most of the researchers in the literature diverted their focus to meta-heuristic methods due to promising results. The solution is obtained either by adopting naturally inspired algorithms, artifacts, or evolutionary techniques. Nature-inspired algorithms generally mimic the phenomena occurring in the nature Wind driven optimization (WDO), Flower Pollination Algorithm (FPA), Grasshopper Optimization Algorithm(GOA), Simulated Annealing (SA), Evaporation-based Water Cycle algorithm (ERWCA), Wind-driven optimization(WDO)^11–16^, while artifact mimics the human intelligence JAYA algorithm, Imperialistic competitive algorithm (ICA), Brain Storming Algorithm (BSA), Drone Squadron Optimization (DSO), Artificial Immune System (AIS), Bio geography-based Optimization (BBO), Harmony Search (HS), Teaching Learning Based Optimization (TLBO) with modifications have evolved^17–24^. Recently Meta heuristic approaches have been used extensively for parameter extraction due to their accuracy and fast computing capability. Researchers have tried to improve the performance by combining multiple algorithms to get the best optimal solution referred to as hybrid algorithms.

An effective parameter estimation method with a hybrid Whale Optimization Algorithm, combined with particle swarm optimization. To improve exploitation capability with reduced range. The error was found to be minimal. WOA adopts the behavior of humpback whales and the searching approach of birds involves PSO exponential function-based PSO with the inertial weight adopted to overcome the problems associated with premature convergence problems^19^. Bacterial foraging optimization involves a greater number of parameters with more computational time^25^. Lambert's function with heuristic adopting differential evolution is proposed to achieve better convergence with a significant reduction in computation time^26^. Mimicking the reproduction behavior of bonobos an optimization algorithm named Bonobo Optimizer (BO) is proposed to demonstrate the efficiency of adopting fusion and fission mechanisms. Bonobos adopt various mating strategies to exploit multidimensional search space to avoid local trapping of the solution. Hence there is an adaptive change in population size^27^. Gradient-based optimization with Eagle Strategy is used to enhance efficiency and robustness.

This paper also evaluates dynamic PV models such as integral and fractional PV solar models. Both static and dynamic models are analyzed to obtain the optimal results^28^. GWO is inspired by food-searching behavior practiced by grasshoppers within the boundary with two different life stages, namely lava for slow exploitation and insect stage for dynamic exploration of the search space^13^. To improve accuracy and reliability, a new change with enhancement in the evolutionary operator is presented using an enhanced Roa-1 algorithm^29^. This approach achieves adaptive population size with a linear minimization strategy. Despite all these algorithms, no single method achieves the least root mean square error considering adaptive climatic conditions for three diode model.

## Numerical modelling frame work

### Three diode modelling by using fifteen parameters

**Figure 2:**
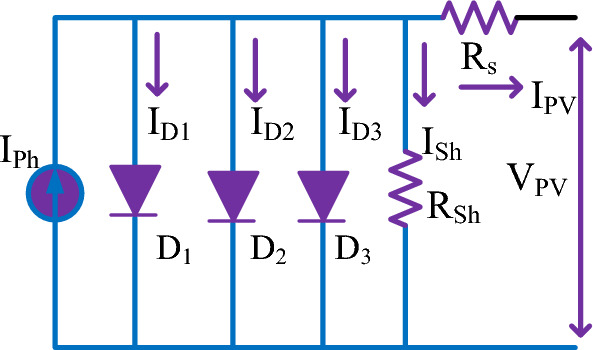
Three diode equivalent model.

There are various models available for performance analysis of solar cells. Modeling of solar cells can adopt single-diode, two-diode, and three-diode approaches. The simplest one-diode model is easy to analyze with compromise in terms of accuracy. Two diode approach is widely used in the literature to extract the parameters it seeks the help of numerical techniques for a few variable extraction and the remaining parameters are estimated using optimization algorithms to reduce complexity. In this manuscript, 3-diode modeling of PV cells is discussed to extract 15 parameters using drone squadron optimization. Here, Fig. 2 presents the 3-diode model of a PV cell consisting of three diode currents I_D1_, I_D2,_ and I_D3_. R_sh_ and R_s_ denote the shunt and series resistances respectively. The photo-generated current is represented by I_ph_, I_PV_ is the output current available to the load and V_PV_ is the voltage measured across the shunt resistance.

1 $${{\text{I}}}_{{\text{pv}}}={{\text{I}}}_{{\text{ph}}}-{{\text{I}}}_{01}\left[{\text{exp}}\left(\frac{{{\text{v}}}_{{\text{pv}}}+{{\text{I}}}_{{\text{pv}}}{{\text{R}}}_{{\text{s}}}}{{{\text{a}}}_{1}{{\text{V}}}_{{\text{t}}}}\right)-1\right]{-{\text{I}}}_{02}\left[{\text{exp}}\left(\frac{{{\text{v}}}_{{\text{pv}}}+{{\text{I}}}_{{\text{pv}}}{{\text{R}}}_{{\text{s}}}}{{{\text{a}}}_{2}{{\text{V}}}_{{\text{t}}}}\right)-1\right]{-{\text{I}}}_{03}\left[{\text{exp}}\left(\frac{{{\text{v}}}_{{\text{pv}}}+{{\text{iI}}}_{{\text{pv}}}{{\text{R}}}_{{\text{s}}}}{{{\text{a}}}_{3}{{\text{V}}}_{{\text{t}}}}\right)-1\right]-\left(\frac{{{\text{v}}}_{{\text{pv}}}+{{\text{I}}}_{{\text{pv}}}{{\text{R}}}_{{\text{s}}}}{{{\text{R}}}_{{\text{sh}}}}\right)$$

2 $${{\text{I}}}_{{\text{ph}}}=\frac{{\text{G}}}{{{\text{G}}}_{{\text{Ref}}}}\left[{{\text{I}}}_{{{\text{ph}}}_{{\text{Ref}}}}+\alpha \left({\text{T}}-{{\text{T}}}_{{\text{Ref}}}\right)\right]$$

3 $${{\text{V}}}_{{\text{t}}}=\frac{{\text{KT}}}{{\text{q}}}$$

4 $${{{\text{I}}}_{{\text{o}}}={\text{I}}}_{{\text{oref}}}{\left(\frac{{\text{T}}}{{{\text{T}}}_{{\text{ref}}}}\right)}^{3}{\text{exp}}\left(\frac{{{\text{qE}}}_{{\text{g}}}}{{\text{aK}}}\left(\frac{1}{{{\text{T}}}_{{\text{Ref}}}}-\frac{1}{{\text{T}}}\right)\right)$$

5 $${{\text{R}}}_{{\text{s}}}={{\text{R}}}_{{\text{s}}2{\text{Ref}}}\left(1+{{\text{k}}}_{{\text{rs}}}({\text{T}}-{{\text{T}}}_{{\text{Ref}}}\right)+{{\text{R}}}_{{\text{s}}1{\text{Ref}}}+{\left(\frac{{\text{G}}}{{{\text{G}}}_{{\text{Ref}}}}\right)}^{{\text{LRS}}}$$

6 $${{\text{R}}}_{{\text{p}}}={{\text{R}}}_{{\text{p}}1{\text{Ref}}}\left(1+{{\text{k}}}_{{\text{rp}}}({\text{T}}-{{\text{T}}}_{{\text{Ref}}}\right)+{\left(\frac{{\text{G}}}{{{\text{G}}}_{{\text{Ref}}}}\right)}^{{\text{LRp}}}$$

Equations to compute output current and photo-generated current under specified irradiation and temperature are given in Eq. (1) and (2). From Eq. (3), T is the temperature K is Boltzmann constant, G is irradiation Where a is the ideality factor, Gref and Tref is the temperature and Irradiance at Standard Test Condition (STC) respectively, Eg is the band gap and q is charge of the electron. From (1), it is evident that there are 15 parameters which are identified as ${{\text{I}}}_{01{\text{ref}}},{{\text{I}}}_{02{\text{ref}}} ,{{\text{I}}}_{03{\text{ref}}}, {{\text{I}}}_{{{\text{ph}}}_{{\text{ref}}} },{{\text{a}}}_{1}, {{\text{a}}}_{2}, {{{\text{a}}}_{3},\text{ L}}_{{\text{Rp}}}, {{\text{L}}}_{{\text{Rs}}},{{\text{K}}}_{{\text{Rs}}},{{\text{K}}}_{{\text{Rp}}},$ ${{\text{K}}}_{{\text{i}}},$ $,{{\text{R}}}_{1{\text{pRef}}},{{\text{R}}}_{{\text{s}}1{\text{Ref}}}\text{ and }{{\text{R}}}_{{\text{s}}2{\text{Ref}}}$. These intrinsic variables exactly determine the performance characteristics of the PV module. Under varying climatic conditions, the open circuit voltage and short circuit current can be determined using the following equation. These intrinsic variables exactly determine the performance characteristics of the PV module. Under varying climatic conditions, the open circuit voltage and short circuit current can be determined using the following Eq’s,

7 $${{\text{I}}}_{{\text{sc}}}={{{\text{I}}}_{{\text{sc}}}}_{{\text{ref}}}+{{\alpha }}_{{\text{i}}}\left(\left({\text{T}}-{{\text{T}}}_{{\text{Ref}}}\right)\right)*\left(\frac{{\text{G}}}{{{\text{G}}}_{{\text{Ref}}}}\right)$$

8 $${{\text{V}}}_{{\text{oc}}}={{{\text{V}}}_{{\text{oc}}}}_{{\text{Ref}}}\left({{\text{V}}}_{{\text{t}}}*{\text{ln}}*\left(\frac{{\text{G}}}{{{\text{G}}}_{{\text{Ref}}}}\right)+{{\alpha }}_{{\text{v}}}({\text{T}}-{{\text{T}}}_{{\text{Ref}}}\right)$$

9 $${\text{f}}\left({{\text{v}}}_{{\text{pv}}},{{\text{i}}}_{{\text{pv}}},{\varnothing }\right)={{\text{I}}}_{{\text{ph}}}-{{\text{I}}}_{01}\left[{\text{exp}}\left(\frac{{{\text{v}}}_{{\text{pv}}}+{{\text{i}}}_{{\text{pv}}}{{\text{R}}}_{{\text{s}}}}{{{\text{a}}}_{1}{{\text{V}}}_{{\text{t}}}}\right)-1\right]-{{\text{I}}}_{02}\left[{\text{exp}}\left(\frac{{{\text{v}}}_{{\text{pv}}}+{{\text{i}}}_{{\text{pv}}}{{\text{R}}}_{{\text{s}}}}{{{\text{a}}}_{2}{{\text{V}}}_{{\text{t}}}}\right)-1\right]-{\text{X}}$$

10 $${\text{X}}={{\text{I}}}_{03}\left[{\text{exp}}\left(\frac{{{\text{v}}}_{{\text{pv}}}+{{\text{i}}}_{{\text{pv}}}{{\text{R}}}_{{\text{s}}}}{{{\text{a}}}_{3}{{\text{V}}}_{{\text{t}}}}\right)-1\right]+\frac{{{\text{v}}}_{{\text{pv}}}+{{\text{i}}}_{{\text{pv}}}{{\text{R}}}_{{\text{s}}}}{{{\text{R}}}_{{\text{sh}}}}+{{\text{i}}}_{{\text{pv}}}$$

11 $$\uptheta =\left\{{{\text{I}}}_{{\text{ph}}},{{\text{I}}}_{01},{{{\text{I}}}_{02,}{{\text{I}}}_{03},{\text{R}}}_{{\text{s}}},{{\text{R}}}_{{\text{sh}}},{{\text{a}}}_{1},{{\text{a}}}_{2}, {{\text{a}}}_{3}\right\}$$

12 $$\text{Normalized RMSE}\hspace{0.17em}=\hspace{0.17em}\left\{\frac{{\text{RMSE}}}{{{\text{I}}}_{{\text{sc}}}}\right\}*100$$

13 $${\text{Min}}\left({\text{F}}\left(\uptheta \right)\right)=\sqrt{\frac{1}{{\text{N}}}}\sum_{{\text{i}}=1}^{{\text{N}}}{{({\text{I}}}_{{\text{i}}}-{{\text{I}}}_{{\text{i}},{\text{ext}}}(\uptheta ))}^{2}$$

where N is the experimental data, I_i_ is the experimental value and I_iext_ denotes the estimated value from 15 parameter extraction. Estimating the parameters for one set of climatic conditions enables the user to predict the power versus voltage (P–V) and current versus voltage (I–V) curves for all weather conditions. This detailed model adapts, despite any variation in radiation and temperature as the intrinsic variables are free to change. This flexibility is not accounted for in classical models available in the literature. On modeling SPV, the precise value obtained by this approach aids in deducing the P–V and I–V curves at varying weather conditions. This enables the industrialist to design a reliable and efficient inverter for any specified location^30^.

## Problem formulation

Global optimization problems are generally derivative-free approaches with no assumptions. A global, auto-adaptive hyper-heuristic algorithm inspired by human Invented drones that can evolve on their own partially. The firmware inside these drones enables the researcher to change the mechanism rather than depending on the bio-inspiration^20^. Despite natural behavior mimicking procedures such as particle swarm optimization. This method inculcates the recombination by varying the solution with a unique procedure to act as a revolutionary approach. In the vast search space, the drones are free to move to explore and exploit the search space. This technique does not inculcate the pre-coded algorithm during its movement, it synthesizes its code to move in a search space. DSO optimization algorithm consists of four major components where the complete task of estimation is carried out. The command center is the most intelligent part where the orders to execute and return drone to the destiny are done by modifying and updating the codes inside the firmware. The perturbation is denoted by P which is the sum of departure and offset. The firmware only produces a trial coordinate (TC) by a perturbation process called biased random walk. This TC is obtained by calculating P. Two arrays are formed, namely current Best and Global Best.

14 $${\text{P}}1:{\text{ GBC}} + \left( {{\text{C}}1 \times \left( {{\text{GBC}} - {\text{CB}}{{\text{C}}_{{\text{drone}}}}} \right)} \right)$$

15 $${\text{P}}2:{\text{ CB}}{{\text{C}}_{{\text{drone}}}} + \left[ {{\text{G}}\left( {0, \, 1} \right) \times \left( {{\text{pU }}\left( {0, \, 1,{\text{ D}}} \right) + {\text{CB}}{{\text{C}}_{{\text{drone}}}}} \right)} \right]$$

where C1 is the constant defined by the user, G the Gaussian distribution, U is the uniform distribution, and D is one of the variables in the objective function which is defined as 15 in this modeling CBC and GBC are current best coordinates and global best coordinates respectively. Offset generally returns the amount of perturbation, thus updation of trial coordinates are computed based on this perturbation function which is denoted by (P). Search space can be shrinked based on departure coordinates which helps to locate the neighboring points. Reference perturbation initializes perturbation to optimize and improve the search performance.

Drone movement stage, the target positions are computed automatically by exploring and exploiting search space using various mechanisms Depending on the choice of recombination trial coordinates, the direction of the drone is fixed to one particular direction. To avoid biasing a correction factor is introduced and the violation limits are perturbed. Firmware update, here, team quality is computed for each iteration to update the rank and violation. The Command Center updates firmware by considering the best and worst by following the rules^20^.

16 $$\text{Violation drone, team }=\sum_{{\text{j}}=1}^{{\text{D}}}({{\text{TmC}}}_{{\text{drone}}},{\text{team}},\text{j }- {{\text{UB}}}_{{\text{j}}})+({{\text{LB}}}_{{\text{j}}}- {{\text{TmC}}}_{{\text{drone}}},{\text{team}})$$

**Figure 3:**
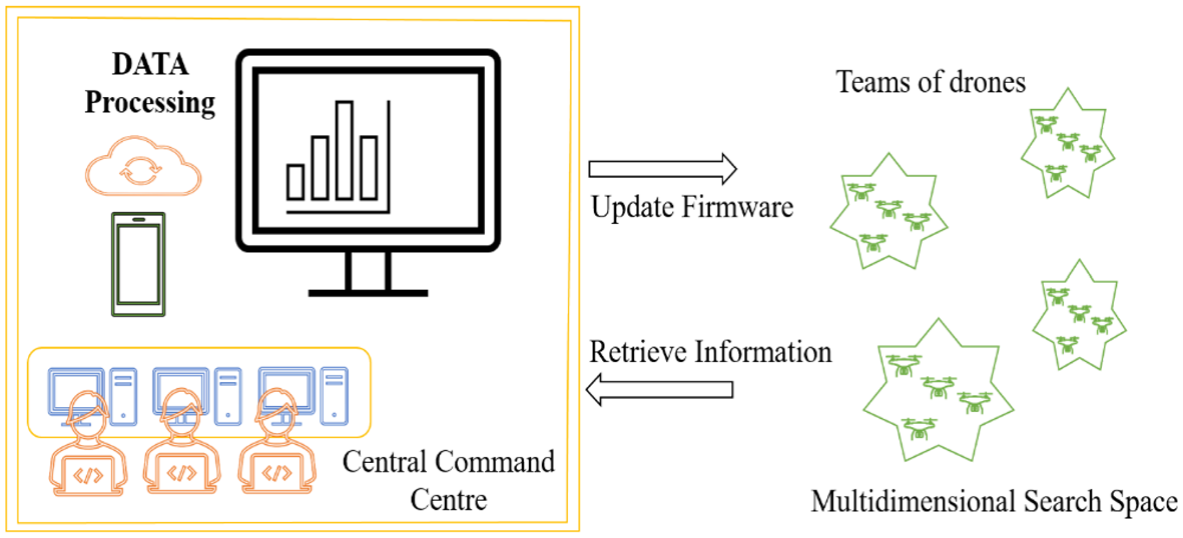
Squadron of drones with central command center for data exchange.

**Figure 4:**
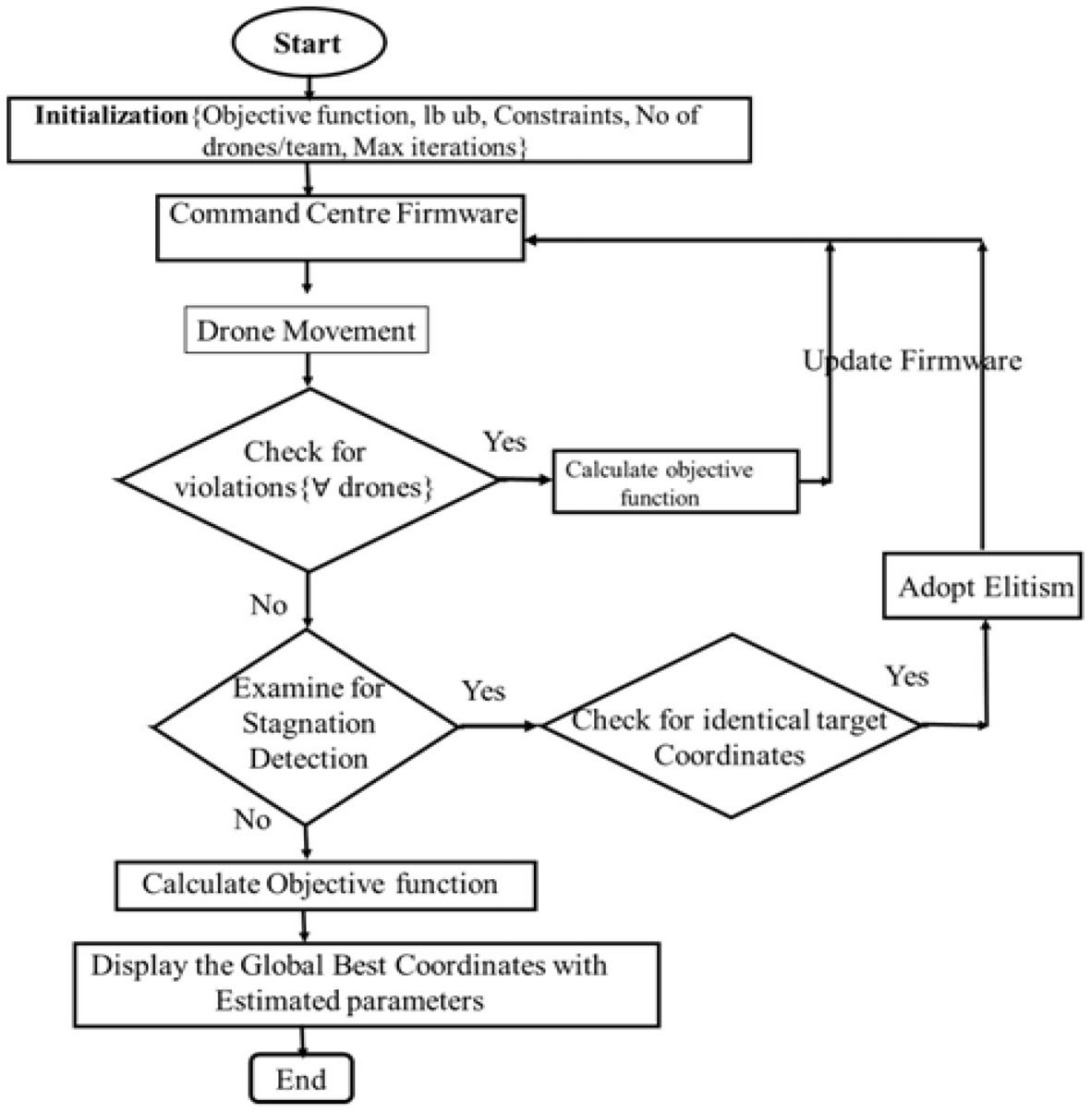
Flow chart depicting process flow diagram of DSO.

Here, UB and LB are upper and lower-bound objective function arrays, and TmC is an array of 2D team coordinates. This violation limit for each team of drones is given in Eq. (15). This violation in each case, for all the drones are considered with the updation of the firmware at the command center accordingly. The stagnation detection and next iteration procedures are repeated until the optimal results are achieved. The flow chart mimicking the pseudo-code is represented in Figs. 3 and 4. It is evident that the root mean squared error estimated for these two cells are 6.7776 × 10^–4^ and estimated for these two cells are 6.7776 × 10^–4^ and 0.231032 × 10^–3^ respectively, which is the least on par with results available in the literature^39^.

## Results analysis

This manuscript proposes a novel DSO-based adaptive algorithm to validate the performance and accuracy of three diode PV model. Two different cases are analyzed to demonstrate accuracy and reliability. In case one, the validation of the proposed approach is carried out considering two commercial PV cells, namely RTC France cell at 33 °C and PWP-201 with 36 cells at Irradiation of 1000 w/m^2^ and temperature of 45 °C using Datasheet values. The Data sheet values specified by the manufacturer are presented in the Table 1. The 15 parameters estimated for the 3-diode model using DSO for two commercial PV cells algorithms are tabulated in Table 2. It is evident that the root mean squared error estimated for these two cells are 6.7776 × 10^–4^ and 0.231032 × 10^–3^ respectively, which is the least on par with results available in the literature^39^.

**Table 1:** Data-sheet values of commercial solar cells.

Cell	RTC France	PWP-201
I_sc_	0.76	1.03
V_oc_	0.5728	16.778
V_mp_	0.45	12.6
I_mp_	0.691	0.898
P_m_	0.311	11.315
T	33	45
N_s_	36	1

**Table.2.** Data-sheet values of commercial solar cells.

Parameters	RTC France	Photowatt-PWP-201
a_1_	2.499932	0.5
a_2_	1.36085097	1.326345746
a_3_	0.500000125	1.326344874
Iph_ref	0.02534768	0.395757676
Ioref1	8.86e-07	7.88e-23
Ioref2	1.33e-09	7.9806e-09
Ioref3	1.09e-25	3.08e-09
Rsref1	0.015035688	1.209706934
Rsref2	0.02305394	0.060448118
Rpref	28.06111293	519.3645624
K_rs_	0.005503801	0.013655225
K_rp_	0.036423602	0.013050909
L_rs_	2.5853434	3.473191096
L_rp_	4.548846404	0.706157287
k_i_	0.022289382	0.014138913

**Figure 5:**
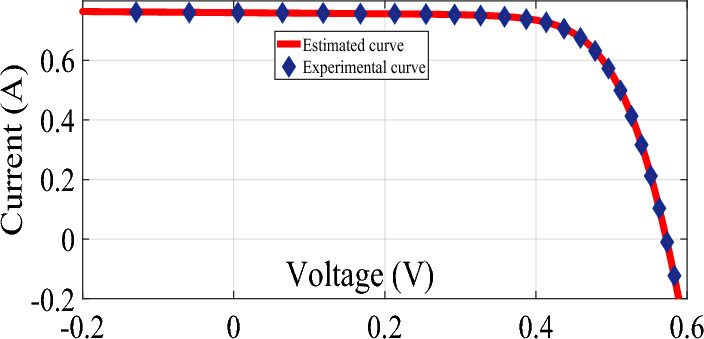
The IV performance characteristics of RTC France Cell with experimental curves overlapping with estimated values.

**Figure 6:**
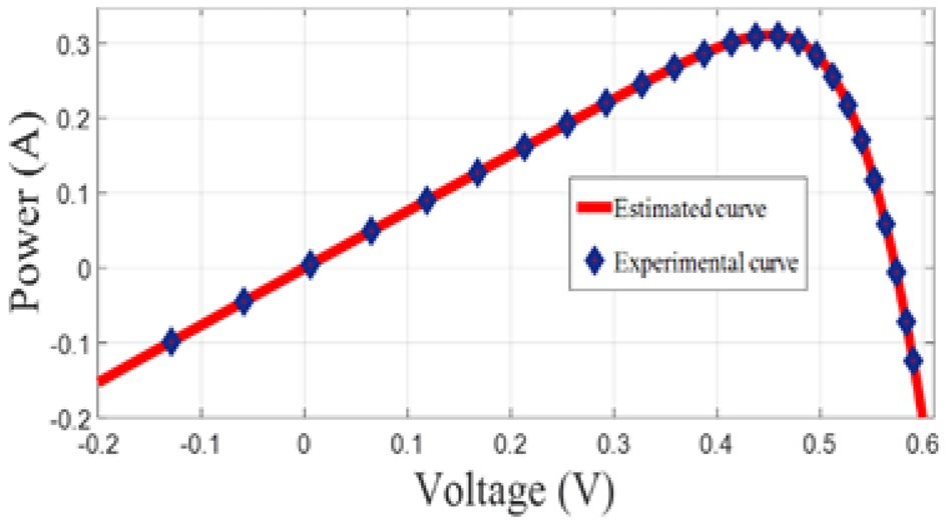
The PV performance characteristics of Photo-watt PWP-201 Cell with experimental curves overlapping with estimated values.

**Figure 7:**
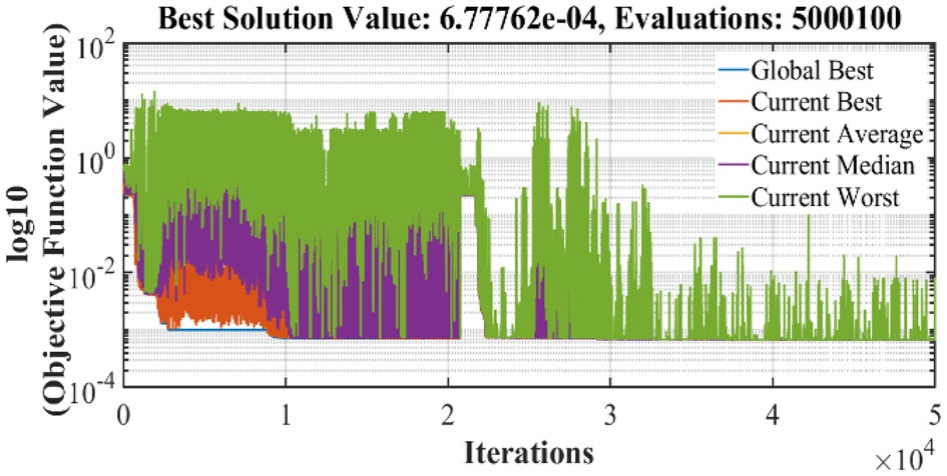
Best run with least RMSE for RTC France Cell.

**Figure 8:**
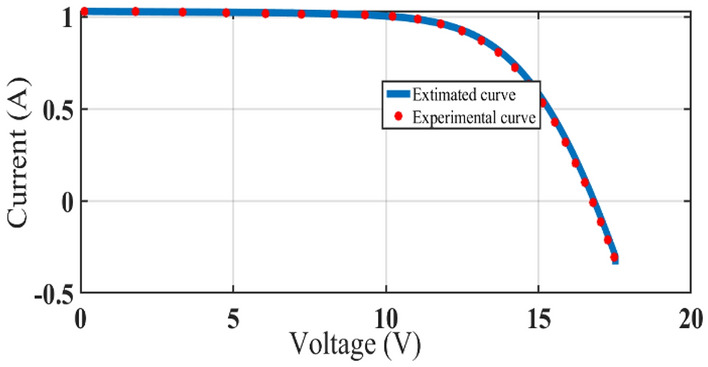
The IV performance characteristics of Photo-watt PWP-201 Cell with experimental curves overlapping with estimated values.

**Figure 9:**
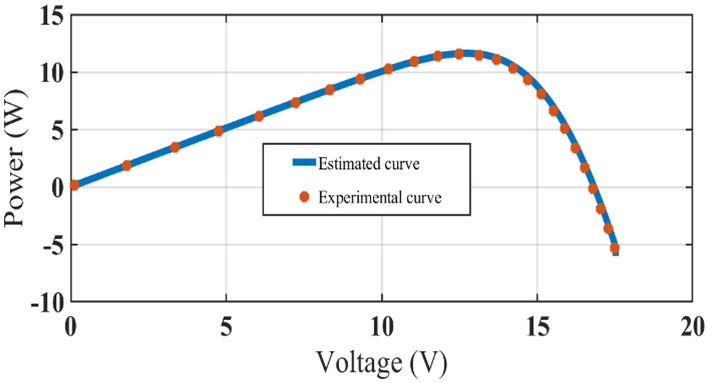
The PV performance characteristics of Photo-watt PWP-201 Cell with experimental curves overlapping with estimated values.

**Figure 10:**
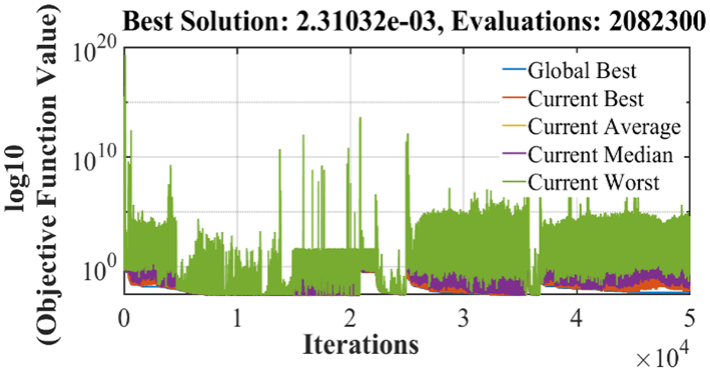
Best run with least RMSE for Photo-watt PWP-201 Cell.

**Figure 11:**
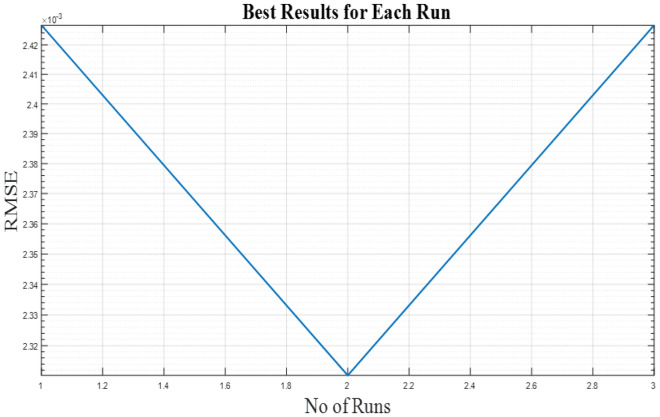
Best results for different runs in terms of RMSE for Photo-watt PWP-201 Cell.

The graphical analysis of RTC France and Photo Watt PWP-201 for PV and IV curves using the DSO algorithm are presented in Figs. 5, 6, 8, and 9. The estimated values coincide with the practical experimental values and hence it exhibits minimum deviation accounting for low RMSE. Figures 7 and 10 show the best objective function with various statistical analysis metrics such as current best, global best, current mean, median, and average for different no of iterations. For each run, the best solution is estimated which is illustrated in Fig. 11. For the RTC France cell the least RMSE of 6.8 × 10^–4^ is obtained with minimal iteration as depicted in the Fig. 8.

Figure 11 presents the best solution of the objective function for two different PV Cells. It is clear from the figure that the solution with the least error is achieved with a minimum no of runs. Table 3 demonstrates the results obtained from the DSO algorithm for the 3-diode model in comparison with eight recent competitive Fig. 11. Presents the best solution of the objective function for two different PV Cells. It is clear from the figure that the solution with the least error is achieved with a minimum no of runs. Table 3 demonstrates the results obtained from the DSO algorithm for the 3-diode model in comparison with eight recent competitive algorithms. In literature^31–36^ parameters are extracted for single and double diode models only. The proposed work estimates 15 parameters of three diode model and comparison is done considering 2 diodes, and 9- parameters. It's worth noting that DSO outperformed all other optimization approaches in terms of attaining the lowest RMSE and fastest execution time while maintaining a strong convergence response. The last row of the table clearly reports the superiority of DSO over other techniques. The low value of RMSE at different temperatures is made bold in Table 3, along with 15 Parameters five other parameters of the module are also estimated to improve the accuracy of the modelling.

**Table 3:** Comparison of estimated parameter of Three diode model of Photo Watt PWP-201 with literature^31^.

Parameters	DSO	AEO ^31^	SSA ^32^	RCGA ^31^	BHCS ^34^	GA ^33^	PSO ^34^	SFO ^35^	CSA ^36^
a_1_	0.5	2.08135	1.9256	1.7945	1.71197	1.7597	1.9848	1	1.4032
a_2_	1.326345746	2.08135	1.7103	1.7162	1.16255	1.398	1.3887	1.4494	1.6116
_a3_	1.326344874	1.36458	1.8094	1.6501	1.28954	1.472	1.8615	2	1.0016
I_ph_	0.862341808	1.03051	1.0388	1.0321	1.0309	1.0267	1.0304	1.0959	1.0297
Io_1_	2.89e-18	0.0023	44.5979	5.222	9.94764	3.1593	1E-12	0.00182	4.981
Io_2_	5.19e-07	0.065	2.06046	34.478	0.000015	5.0722	4.8769	4.4985	2.6813
Io_3_	2.00e-07	1.1893	56.048	8.6879	1.51074	4.1141	1.1269	9.9896	8.2227
Rs	1.297	1.0154	0.917	0.86	1.4149	1.163	1.1638	3.1469	1.1308
R_p_	743.044	1.91858	85.67	180	1.0214	16.479	1.2483	14.653	1.809
RMSEx10^–4^	0.002310324	0.00248	0.0135	0.0153	0.003679	0.0037	0.0027	0.0825	0.0032

In case two, the DSO approach has been implemented on a large-scale system to investigate the performance using a 3-diode model under different irradiation and temperature. The experimental data from the PV array is used in this case study. Three strings are linked in parallel with six strings in the section of the PV array that is being used per string, there are PV modules. The PV module's identifier is a mono-crystalline GL-M100 made up of 36 cells in a single crystal series. Temperature and irradiance are the I–V characteristics. The PROVA1011 I–V tester was used to measure the results. The PV module's electrical properties are accessible in^37^.

To demonstrate, a single-diode model was used. To investigate the performance of the suggested algorithm, the approach has been put into practice on a wide scale. The experimental values of PROVA1011 are also taken into consideration to evaluate the iv characteristics under real-time changes in irradiation and temperature. The graph in Fig. 11. demonstrates the accuracy of estimated values using the DSO optimization technique with the experimental curves. A comparison with the literature^38^ is carried out to demonstrate the effectiveness of the DSO algorithm. RMSE estimated using the DSO approach shows superior results as compared with recent literature. This work aims for reliable estimation of parameters since it accounts for all the changes concerning irradiation and temperature. The detailed parameters estimation of the 3-diode model solar system is given in Table 4.

**Table 4:** Parameters estimated for three diode models with different irradiation and temperature.

	DSO	ABC-TRR^38^	DSO	^38^	DSO	^38^	DSO	^38^	DSO	^38^	DSO	^38^
Parameters	G = 553; T = 41.4;		G = 551; T = 52.5;		G = 442; T = 36.7;		G = 390; T = 35.9		G = 333; T = 32.4		G = 281; T = 30.3	
RMSE	0.0579	0.058	0.0544	0.058	0.0313	0.0313	0.029	0.0291	0.0181	0.0182	0.013336	0.0134
Iphref	10.007	NA	10.6638	NA	4.301	NA	5.668	NA	8.427	NA	9.83578	NA
Ioref1	0	NA	0	NA	3.78e-07	NA	7.744	NA	1.15e-10	NA	9.41e-71	NA
Ioref2	1.33e-09	NA	0	NA	0	NA	0	NA	9.74e-10	NA	1.52e-31	NA
Ioref3	0	NA	1.61e-09	NA	9.89e-10	NA	1.64e-09	NA	0	NA	1.27e-09	NA
N1	5.6105	5.996	9.0165	6.467	9.9724	230.29	9.151	225.7	6.2707	225.75	0.8798	228.8
N2	5.99638	NA	2.2852	NA	6.4739	NA	2.686	NA	6.2707	NA	4.3884	NA
N3	6.64588	NA	5.97750	NA	6.11363	NA	6.271	NA	6.97470	NA	0.3978	NA
Rsref1	0.02545	NA	1.4092	NA	0.4868	NA	0.7521	NA	0.72375	NA	5.1421	NA
Rsref2	2.04332	NA	0.0599	NA	0.4210	NA	1.5182	NA	0.94230	NA	0.318	NA
Rpref	599.356	NA	619.696	NA	422.83	NA	222.13	NA	28.9716	NA	59.913	NA
Krs	0.00369	NA	0.39525	NA	0.1036	NA	0.0089	NA	0.04715	NA	0.002	NA
Krp	0.00267	NA	0.00391	NA	0.0261	NA	0.0508	NA	0.7670	NA	0.329	NA
rs	0.218986	NA	0.21512	NA	0.1900	NA	0.201	NA	0.95802	NA	0.6415	NA
rp	0.9995	NA	0.95416	NA	0.77255	NA	0.2097	NA	0.23214	NA	0.0243	NA
ki	0.43594	NA	0.11628	NA	7.99855	NA	0.3460	NA	0.29776	NA	0.272	NA
Iph	10.0043	10.0007	9.23795	8.0012	7.99855	8	7.0551	7.06	6.01895	6.02	5.0813	5.08
Io1	0	0.057e-06	0	0.01276	9.65e-07	0.0099	2.04004	0.0057	3.58e-10	0.0034	2.73e-68	0.0032
Io2	5.72e-09	NA	0	NA	0	NA	0	NA	3.03e-09	NA	6.10e-31	NA
Io3	0	NA	9.68e-09	NA	3.60e-09	NA	5.7e-09	NA	0	NA	3.65e-09	NA
Rs	2.69915	2.699	2.54461	2.5456	2.591345	2.568	2.6297	2.63	2.63445	2.634	2.6219	2.62
Rp	368.2330	368.2	423.119	426.176	441.2868	419.8	515.45	515.5	580.2417	580.2	638.34	621.8

## Conclusion

Three-diode modeling poses a complexity due to the inclusion of various parameters. Single and double-diode models are most commonly used to estimate the five and seven parameters due to simplicity. An attempt towards addressing the complex Two cases with three different commercial solar cells/modules were considered to test the accuracy of the result. Real-time experimental data under changing irradiation and temperature conditions are also considered to check the effectiveness of the proposed DSO algorithm. Two cases with three different commercial solar cells/modules were considered to test the accuracy of the result. Real-time experimental data under changing irradiation and temperature conditions are also considered to check the effectiveness of the proposed DSO algorithm.

A comprehensive comparison of recent Meta-heuristic algorithms with recent literature is illustrated to prove the accuracy and reliability of the proposed work. Despite modeling complexities, this work ensures optimal results with good precision. This DSO can further be used to solve complex multi-objective optimization problems in fields of renewable energy, power systems, and smart grids. This research work enables budding researchers who study the impact of partial shading and charming, as it accounts for various parameters and constraints. A high level of precision between the estimated values and experimental values indicates the robustness of modeling and its adaptation to the DSO algorithm. The obtained result proves the superiority of the algorithm in extracting the PV parameters accurately.

## Data Availability

The data used to support the findings of this study are included in the article.
